# How to induce protective humoral immunity against *Plasmodium falciparum* circumsporozoite protein

**DOI:** 10.1084/jem.20201313

**Published:** 2022-01-10

**Authors:** Ilka Wahl, Hedda Wardemann

**Affiliations:** 1 B Cell Immunology, German Cancer Research Center, Heidelberg, Germany

## Abstract

The induction of protective humoral immune responses against sporozoite surface proteins of the human parasite *Plasmodium falciparum* (*Pf*) is a prime goal in the development of a preerythrocytic malaria vaccine. The most promising antibody target is circumsporozoite protein (CSP). Although PfCSP induces strong humoral immune responses upon vaccination, vaccine efficacy is overall limited and not durable. Here, we review recent efforts to gain a better molecular and cellular understanding of anti-PfCSP B cell responses in humans and discuss ways to overcome limitations in the induction of stable titers of high-affinity antibodies that might help to increase vaccine efficacy and promote long-lived protection.

## Teaser

Antibodies against *Plasmodium falciparum* circumsporozoite protein (PfCSP) can protect from malaria. However, the efficacy of anti-PfCSP vaccine responses is limited. This review summarizes our molecular and cellular understanding of humoral anti-PfCSP immune responses and discusses implications for vaccine design.

## Introduction

Malaria is a curable mosquito-transmitted disease caused by unicellular *Plasmodium* parasites with large genomes that have developed complex life cycles during the long coevolution with their human host. Extensive prevention, diagnosis, and treatment measures have helped to reduce the number of malaria cases over the past decades (World Health Organization, 2020). However, with 229 million cases in 2019, the global disease burden is still high, and the emergence of insecticide-resistant mosquitoes, multidrug-resistant parasites, and deletion mutants that lack biomarker genes for rapid diagnostic tests is a threat to the global control and eradication strategy. The negative impact of the SARS-CoV-2 pandemic on the delivery of medical and nonmedical malaria services complicates the situation and might halt or even reverse the achievements that have been made in many of the most affected endemic countries over the past years (Sherrard-Smith et al., 2020).

**Figure 1.**
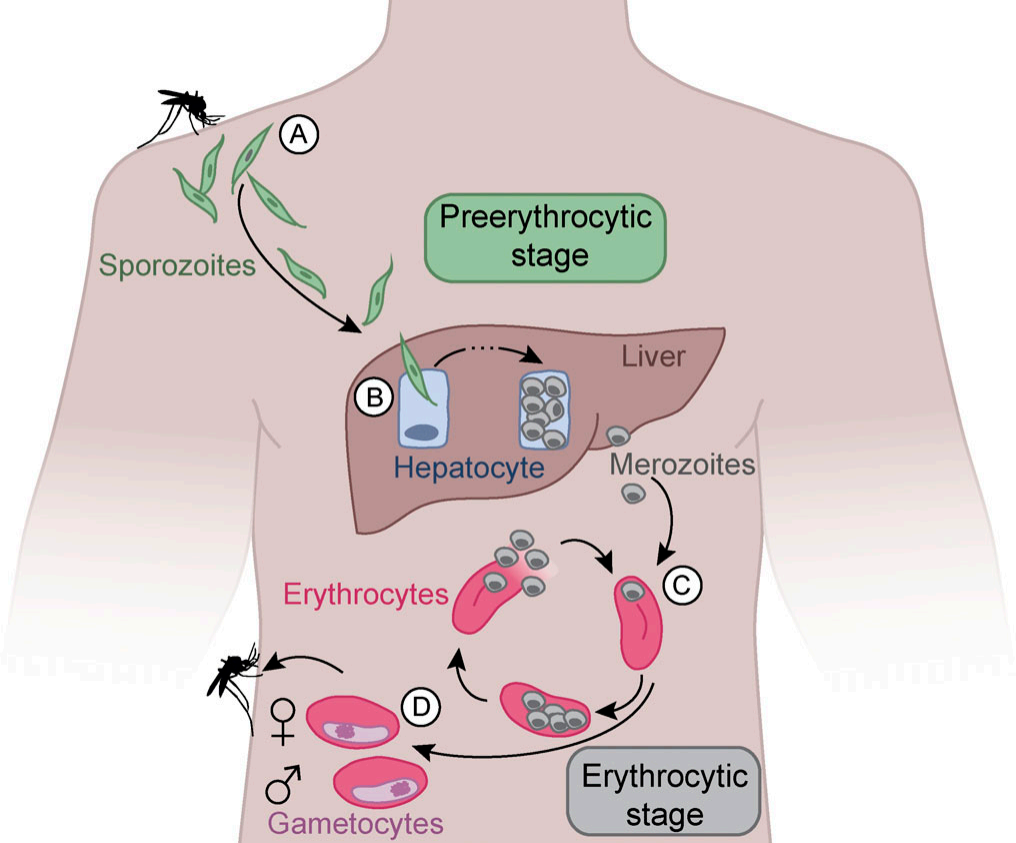
**Life cycle of *Pf*.** **(A)** During the blood meal of an infected *Anopheles* mosquito, sporozoites are injected into the human skin. **(B) **Sporozoites migrate through the blood stream to the liver and infect hepatocytes. In hepatocytes, the parasite multiplies and differentiates into asexual merozoites that rupture the infected host cell and enter the blood stream. **(C)** In the blood, merozoites infect erythrocytes for repeating rounds of asexual replication, erythrocyte lysis, and infection of fresh erythrocytes, resulting in exponential parasite growth associated with symptom onset and malaria disease. **(D)** Some merozoites differentiate into female and male gametocytes. These sexual erythrocytic parasites can be taken up by a mosquito during another blood meal for completion of the life cycle in the insect vector.

Of the five *Plasmodium* species that infect humans, *Plasmodium falciparum* (*Pf*) causes the most life-threatening form of malaria. Its prevalence is highest in Sub-Saharan Africa, a region that carries nearly 95% of global malaria cases and deaths, with the vast majority among children below the age of 5 (World Health Organization, 2020). Presumably due to the low parasite burden, the first preerythrocytic phase of the *Pf* life cycle is asymptomatic (Fig. 1; Cowman et al., 2016). During a blood meal, infected female *Anopheles* mosquitoes transmit a very small number of *Pf* sporozoites, which reside in their salivary glands, into the human skin. The parasites quickly enter the circulation to invade the liver and infect hepatocytes for asexual multiplication and development into merozoites. The symptomatic erythrocytic phase of the disease starts ∼1 wk later, when large numbers of these blood-stage parasites are released into the circulation and start to invade and multiply in erythrocytes in repeating cycles. Some of the parasites in infected erythrocytes develop into male or female gametocytes that if transmitted to a mosquito during a blood meal can complete the parasite life cycle in the insect vector. While the sexual blood stages, which represent only a minor fraction of erythrocytic parasites, are not associated with disease symptoms, the fast cyclic growth of the merozoite population and associated erythrocyte lysis induce strong inflammatory responses and fever. Due to changes in their shape and stiffness, the infected cells are readily filtered by the spleen. However, the overall loss of erythrocytes can lead to severe anemia, especially in young children. Additional life-threatening complications are caused by alterations in the cytoadhesive properties of the infected erythrocytes that mediate their sequestration in microvessels, e.g., in the brain or lung, to avoid splenic clearance (Jensen et al., 2020). Pregnancy increases the likelihood of developing severe disease and poses a great risk to the mother and her unborn child. Early diagnosis and immediate drug treatment are key to prevent the fast progression from mild to severe symptoms, such as cerebral malaria or multiorgan failure.

Although cellular immune mechanisms likely contribute to parasite control (Kurup et al., 2019), a major goal in malaria research has been the development of subunit vaccines that elicit protective humoral immune responses against *Pf*. Numerous candidates against target antigens on sporozoites, merozoites, and gametocytes have been designed to prevent *Pf* infection, disease, and vector transmission, respectively (Duffy and Patrick Gorres, 2020). Here, we discuss possible limitations of success and the latest developments in the rational design of a malaria subunit vaccine, with a special focus on strategies that aim at preventing *Pf* infections through the induction of humoral immune responses against sporozoites.

## Vaccine approaches targeting erythrocytic parasites

Although untreated primary *Pf* infections are life threatening in all age groups, repeated exposures to *Pf* induce a state of asymptomatic immune control that is characterized by limited blood-stage parasitemia and balanced inflammatory immune reactions (Doolan et al., 2009). Humoral immunity plays a major role in controlling blood-stage parasites, as demonstrated by historic passive transfer experiments with serum antibodies (Cohen et al., 1961; Edozien et al., 1962). Targets of inhibitory antibodies are known merozoite surface antigens, including members of the erythrocyte invasion complex and parasite antigens on the surface of infected erythrocytes, and likely proteins whose role in parasite biology has not been determined (Davies et al., 2015; Tuju et al., 2017). The antibodies mediate their function by opsonization, complement activation, and agglutination and can interfere with the host cell invasion process or cytoadherence and sequestration of infected erythrocytes (Hill et al., 2017; Lu et al., 2018; Kurtovic et al., 2020). Several asexual blood-stage antigens have been explored as vaccine targets to prevent malaria (Duffy and Patrick Gorres, 2020). The overall limited success of these efforts has been linked to sequence polymorphisms and functional redundancies, strategies that help the parasite to evade host immune responses, and the overall large number of antigens.

More recently, sexual stage *Pf* antigens have been explored for the design of vaccines that aim at preventing parasite transmission and development in the mosquito. Similar to the antimerozoite response, natural parasite exposure induces humoral immune responses against sexual stage antigens (de Jong et al., 2020). The antibodies can impair human-to-mosquito transmission by reducing the numbers of sexual stage–infected erythrocytes or direct interference with parasite development in the mosquito midgut upon egress from the infected erythrocyte (de Jong et al., 2020). For a handful of sexual stage antigens, vaccine candidates have been developed (Duffy and Patrick Gorres, 2020). Their ongoing clinical development will show whether the induced immune responses can truly block parasite transmission to the vector or reduce the number of developing sporozoites sufficiently to interrupt the life cycle and prevent infection of the human host.

## Preerythrocytic vaccines—RTS,S and beyond

**Figure 2.**
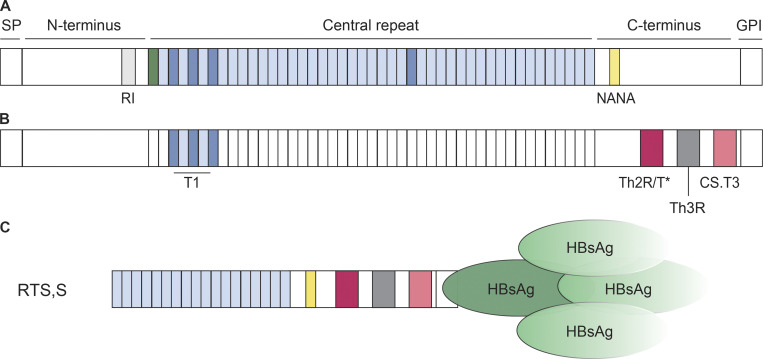
**Schematic representation of PfCSP and RTS,S.** **(A)** PfCSP consists of a signal peptide (SP), an N-terminal domain with the RI, a central repeat region, and a C-terminal domain. It is anchored in the sporozoite membrane through a GPI anchor. The N-terminal domain is linked to the central repeat region, composed of repeating NANP motifs (light blue), via a junctional region with a single NPDP (green) followed by a small number of NANP and alternating NVDP (dark blue) motifs. Protective antibodies recognize these four aa motifs and a single NANA (yellow) motif in the C-terminal domain (Julien and Wardemann, 2019). **(B)** Known T cell epitopes in the junctional region (T1; Nardin et al., 1989) and the C-terminal domain (Th2R/T*, Th3R, CS.T3; Good et al., 1988; Guttinger et al., 1988; Sinigaglia et al., 1988a; Moreno et al., 1991) are highlighted in colors. **(C)** RTS,S consists of 18 NANP repeats and the complete PfCSP 3D7 C-terminal domain fused to HBsAg and complexed with free HBsAg in a 1:3 ratio.

Due to the low number of injected sporozoites, this stage represents a bottleneck in the parasite life cycle. Although natural infections induce only weak nonprotective antisporozoite immune responses, repeated exposure to high doses of chemically, genetically, or radiation-attenuated *Pf* sporozoites can mediate protection (Goh et al., 2019). T cell– and natural killer (NK) cell–mediated immune mechanisms likely contribute to parasite control under these conditions, but their role in protection is not fully understood (Kurup et al., 2019; Goodier et al., 2020). Humoral immune responses target numerous sporozoite antigens, a small number of which have been assessed as vaccine targets in clinical trials (Trieu et al., 2011; Bettencourt, 2020). Of these, only the main sporozoite surface protein circumsporozoite protein (CSP), a glycosylphosphatidylinositol (GPI)-anchored protein with three domains (N-terminal, repeat, C-terminal) that densely coats the sporozoite membrane (Dame et al., 1984), showed efficacy as a vaccine target and induced antibody titers linked to protection (Fig. 2; McCall et al., 2018). Research on CSP began several decades ago and quickly led to vaccine development efforts based on the observation that antibodies against the central domain, characterized by repeating asparagine-alanine-asparagine-proline (NANP) motifs in *Pf*, mediated protection in animal models (Cohen et al., 2010). The fact that the NANP motifs are 100% conserved in all *Pf* parasites and that CSP is essential for parasite development in the mosquito vector and mammalian host, as shown for rodent *Plasmodium* species, supported this choice. RTS,S, the only *Pf* malaria vaccine that ever completed a clinical phase III trial successfully, targets PfCSP (Agnandji et al., 2012). RTS,S was designed to contain 18 repeating NANP motifs (R) in the central repeat domain and the complete PfCSP C-terminus covering the vast majority of known T cell epitopes (T) but not the N-terminus or the junction between the N-terminus and central domain that contains alternating variant or minor repeat (asparagine-proline-aspartate-proline [NPDP], asparagine-valine-aspartate-proline [NVDP]) and NANP motifs (Fig. 2, B and C). Immunogenicity was boosted by genetic fusion with hepatitis B surface antigen (HBsAg; S) and complexation with an excess of soluble HBsAg, promoting self-assembly into virus-like particles (RTS,S). Unfortunately, in the final field study, RTS,S in AS01, a liposome-based adjuvant, showed limited efficacy and relatively short-lived protection (Agnandji et al., 2012). Recent data suggest that delayed booster immunizations with lower doses might lead to better immune responses and protection (Regules et al., 2016; Moon et al., 2020; Pallikkuth et al., 2020). However, these regimens have not been tested in field trials or children. More recently, R21 has been developed, which resembles RTS,S but does not contain non-PfCSP–coupled HBsAg to focus the response on the protective PfCSP rather than on the nonprotective HBsAg epitopes (Collins et al., 2017). Indeed, emerging data from a phase II field trial with R21 in matrix M, a lipid particle–based adjuvant, suggest that R21 shows improved immunogenicity and protection (Datoo et al., 2021), but future studies will have to confirm these findings and show whether this is a durable effect and to what extent it depends on the difference in adjuvant rather than immunogen design. Given the relative success of RTS,S and R21, PfCSP remains a prime malaria vaccine target (Langowski et al., 2020; Friedman-Klabanoff et al., 2021; Reuling et al., 2020). However, the question arises how can we design an improved PfCSP-based immunogen that induces better protection and more durable immune responses against *Pf* malaria? The efficacy of humoral immune responses depends not only on the quantity but also on the quality of serum antibodies, which is strongly defined by their target epitope specificity and binding affinity. Strong germinal center (GC) reactions are essential to drive the continuous generation and selection of antibody variants with improved affinity and underlie the formation of stable memory responses. Thus, from a basic immunology perspective, improving the quality rather than quantity of the humoral response may be key to success. Recent insights from the functional anti-PfCSP B cell and T cell repertoire analyses in humans that we describe below provide a starting point for the rational design for a second-generation PfCSP-based subunit vaccine.

## Specificity, quality, and function of anti-PfCSP antibodies

mAbs are powerful tools to define the precise target epitopes of protective humoral immune responses at the molecular level and to aid the design of rational vaccines, i.e., against malaria (Kwong and Mascola, 2012; Julien and Wardemann, 2019; Graham et al., 2019). Over the past years, numerous human mAbs against PfCSP have been characterized at the molecular and functional level to identify the most promising target epitopes beyond the well-described NANP motifs (Julien and Wardemann, 2019). The antibodies were obtained by Ig gene expression cloning from B cells of malaria-naive volunteers after i.v. injection of high doses of radiation or chemically attenuated sporozoites with a developmental liver or asexual blood-stage block, respectively; from B cells of malaria-exposed donors undergoing controlled human malaria infections with low doses of fully infectious sporozoites; and from RTS,S vaccinees (Oyen et al., 2017; Triller et al., 2017; Kisalu et al., 2018; Murugan et al., 2018; Scally et al., 2018; Tan et al., 2018). Based on passive transfer experiments in mice and challenge with PfCSP-expressing rodent *Plasmodium berghei* parasites, the studies showed that the most potent antibodies recognized the central repeat domain or the junction between the N-terminus and central repeat domain, which contains a single NPDP motif followed by a small stretch of alternating NANP and NVDP motifs and is not contained in RTS,S (Fig. 2 A; Kisalu et al., 2018; Tan et al., 2018; Murugan et al., 2020; Oyen et al., 2020). High affinity was associated with cross reactivity to these junctional motifs and flexibility in accommodating the aa sequence variants, including a unique NANA motif in the C-terminus (Murugan et al., 2020). Although affinity rather than preferential binding of cross-reactive antibodies to the central repeat or the junction seems to be linked to parasite inhibition, this association may not be absolute. A recent landmark study provided first evidence that passive immunization with a potent anti-PfCSP mAb, a modified version of the anti-junction mAb CIS43 with extended half-life, can mediate full protection from *Pf* infection (Gaudinski et al., 2021; Kisalu et al., 2021). These first proof-of-concept results encourage ongoing and future studies with more candidate antibodies to define the relationship between antibody fine specificity, titer, and protective efficacy, especially in the field.

The few reported antibodies with specificity for the PfCSP C-terminal domain failed to bind sporozoites and lacked inhibitory activity, presumably due to inaccessibility of the domain on the sporozoite surface (Scally et al., 2018). Strikingly, antibodies with reactivity to the N-terminus upstream of the conserved region I (RI) were not reported in any of the human studies, likely reflecting the overall poor immunogenicity of the N-terminal domain also seen at the polyclonal level or absence of the N-terminus due to cleavage in the mosquito (Coppi et al., 2005; Herrera et al., 2015; Kisalu et al., 2018; Murugan et al., 2018; Tan et al., 2018, 2021; Cawlfield et al., 2019). Nonetheless, the fact that a monoclonal mouse antibody with high affinity against RI lacked sporozoite reactivity and protective efficacy suggests that the N-terminus might not be a promising vaccine target (Thai et al., 2020). Thus, the number of known PfCSP epitopes that are recognized by protective antibodies is limited to NANP and NANP-variant motifs in the central repeat domain and N-terminal junction.

The exact mechanisms by which these antibodies block parasite development are still poorly understood. The major function of anti-CSP antibodies seems to be the precipitation of the protein from the parasite surface, thereby inhibiting parasite motility and egress from the inoculation site in the skin, as well as neutralizing sporozoites in the liver (Aliprandini et al., 2018; Wang et al., 2020). However, it remains to be determined to what extent parasite inhibition occurs in skin, circulation, and liver and whether antibodies differ in their mode of action or effectiveness in different anatomical locations. A better understanding of the molecular mechanism underlying antibody–PfCSP interactions on live sporozoites in vitro and in vivo in the tissue context of the mammalian host will help to identify antibodies that mediate effective protection after passive immunization.

The potency of anti-PfCSP antibodies may also be affected by their isotype or subclass and associated effector function, e.g., via complement fixation and interactions with Fc receptors mediating phagocytosis or parasite killing by NK cell activation (Leitner et al., 2020). Numerous serological studies investigated the link of these antibody properties to protection (Suscovich et al., 2020; Seaton et al., 2021; Young et al., 2021). However, the polyclonal composition of serum antibodies, the fact that antibody specificity and effector function are directly linked, and differences in assay setups across studies complicate these analyses. Assessments of RTS,S AS01–induced responses in two controlled human malaria infection trials with malaria-naive adults have reported antibody-dependent cellular phagocytosis at the day of parasite exposure as a predictive immune measure of protection along with anti-NANP titers (Young et al., 2021). Unfortunately, our immunological toolbox to direct class–switch recombination and antibody effector function of vaccine responses toward antibody-dependent cellular phagocytosis is largely limited to the empirical use of adjuvants and delivery platforms. Thus, the most promising way forward in the design of a second-generation PfCSP vaccine is the inclusion of the protective junctional epitopes that are not contained in RTS,S or R21 (Cawlfield et al., 2019; Atcheson et al., 2021; Francica et al., 2021; Jelínková et al., 2021). Studies to identify vaccine formulations that induce protective antibodies with optimal effector functions should follow.

## Anti-PfCSP B cell memory formation

Besides the identification of protective anti-PfCSP antibodies and the molecular characterization of their target epitopes, a deep understanding of the cellular origin and maturation pathway of these antibodies is important for the development of a vaccine that induces high-quality B cell responses. A major goal in vaccine design is the efficient recruitment of B cells that express the most potent antibodies to drive their affinity maturation in GC reactions and induce efficient B cell memory formation for long-term protection not seen with RTS,S (White et al., 2015). Longitudinal single-cell–based analyses have shed light on the cellular origin and clonal evolution of the anti-PfCSP B cell response (Triller et al., 2017; Murugan et al., 2018). Although highly mutated, preexisting memory B cells and naive B cells with low PfCSP reactivity seeded the response after sporozoite immunization, naive B cells expressing potent *IGHV3-33*–encoded anti-NANP antibodies dominated the antiparasite response at later time points (Murugan et al., 2018). Experimental evidence and computational modeling showed that the recruitment of high PfCSP-binding B cells from the naive pool rather than the selection of emerging clonal variants in GC reactions drove affinity maturation of the anti-PfCSP response at the population level.

Potent *IGHV3-33*–encoded anti-PfCSP antibodies were identified in several independent studies (Imkeller et al., 2018; Murugan et al., 2018; Oyen et al., 2018; Tan et al., 2018; Wang et al., 2020). The strong enrichment of VH3-33 antibody–expressing cells was linked to a germline-encoded tryptophan at position 52 of IgH complementary determining region 2 with a key role in NANP binding that is not encoded by highly similar *IGHV* gene segments (Imkeller et al., 2018; Murugan et al., 2020). Structural polymorphisms in the *IGH* locus might explain interindividual differences in the frequency of VH3-33 cells, including their complete lack in individuals without the corresponding gene segment, but it remains to be determined how this might affect vaccine efficacy in human populations (Watson et al., 2013). Although VH3-33 antibodies frequently show germline reactivity to PfCSP, especially in combination with VK1-5 light chains and short κ-complementary determining region 3, clonal variants with improved reactivity developed over time (Murugan et al., 2020). Strikingly, the mutations improved antigen binding not only through mutations at the paratope–antigen interface but also by mediating interactions between two neighboring antigen-bound antibody molecules, enabled by the repetitive nature of the target epitopes that characterize the PfCSP repeat domain (Imkeller et al., 2018; Oyen et al., 2018). These homotypic antibody–antibody interactions, formed by VH3-33 and non–VH3-33 anti-PfCSP antibodies, enable dense packing of antibody molecules on recombinant PfCSP, and do not require somatic mutations (Kucharska et al., 2020; Pholcharee et al., 2021), but whether antibodies engage in homotypic interactions on the parasite surface remains to be determined. Positive effects of this binding mode on parasite inhibition have not been reported so far (Imkeller et al., 2018). However, evidence exists that homotypic antibody–antibody interactions promote B cell activation in response to stimulation with antigens containing repeating NANP epitopes that enable B cell receptor cross linking. How this novel affinity maturation process influences the selection and differentiation of B cells expressing antibody variants with improved direct antigen binding during GC reactions remains to be determined.

Homotypic interactions might, however, also have a negative impact on antibody titers, as the fast development of antibody-secreting cells and production of serum antibodies that bind with high avidity to the repeating epitopes in the central PfCSP domain may feed back negatively on the development, selection, and differentiation of high-affinity B cells (Fisher et al., 2017; Toellner et al., 2018; McNamara et al., 2020; Chatterjee et al., 2021). Long immunization schedules with low-dosing regimens might help to promote GC responses over short-lived plasma blast responses and foster the selection of somatically mutated antibody variants, including those with strong binding to junctional epitopes that require time to develop (Murugan et al., 2018). Thus, improving the quality rather than the quantity of the anti-PfCSP B cell response might help in the development of a vaccine with higher efficacy.

## Importance of T cell help

**Figure 3.**
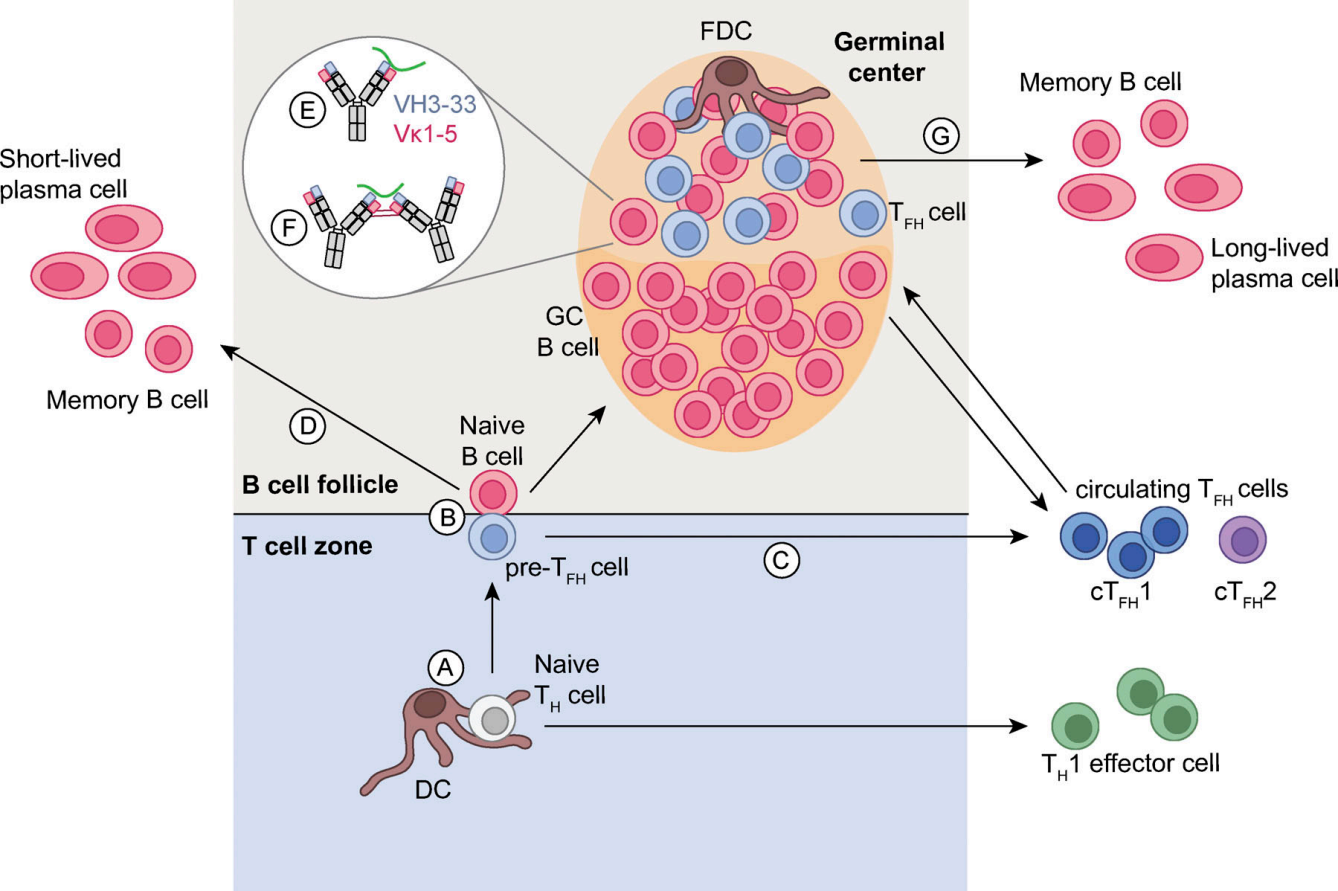
**Schematic overview of the activation and differentiation pathways of human B cells targeting PfCSP.** **(A) **Naive CD4^+^ Th cells are primed by dendritic cells (DCs), which process and present PfCSP antigens on MHC-II molecules in the T cell zone of secondary lymphoid organs (SLOs). **(B) **Naive CD4 Th cells differentiate into Th1 effector cells and exit the SLO or differentiate into precursor T_fh_ cells (pre-T_fh_) and migrate to the border of the T cell zone and the B cell follicle, where they interact with B cells that have been activated by antigen. **(C and D)** Antigen-activated B cells and pre-T_fh_ cells differentiate into memory B cells and short-lived plasma cells or cT_fh_ cells, respectively, and exit the SLOs or migrate into the B cell follicle to form GCs. In GCs, B cells undergo affinity maturation upon the induction of somatic hypermutations followed by competition for antigen presented on follicular dendritic cells (FDCs) and survival signals from T_fh_ cells after antigen uptake and presentation on MHC-II molecules. **(E and F)** PfCSP-reactive B cells can undergo either classical affinity maturation (E) by acquisition of mutations that enhance interactions with the antigen or by homotypic affinity maturation (F) through the selection of mutations that facilitate the interaction with B cell receptors bound to neighboring epitopes on the same antigen, e.g., B cell receptors with Ig chains encoded by IGHV3-33 and IGKV1-5 gene segments that recognize NANP motifs in PfCSP (Imkeller et al., 2018; Oyen et al., 2018). **(G) **Upon positive selection, affinity-matured B cells differentiate into memory B cells or plasma cells, some of which exit the SLO, home to the bone marrow, and become long lived. A fraction of GC T_fh_ cells exits the SLO, enters the circulation, and predominantly adopts a Th1-like phenotype characteristic for *Pf* infection (Obeng-Adjei et al., 2015; Chan et al., 2020).

Key to the success of PfCSP-based vaccines might be the development of long-lived affinity-matured plasma cells that maintain protective antibody titers to target sporozoites in the short time window of only minutes to a few hours before the invasion of hepatocytes. T follicular helper (T_fh_) cells play a key role in B cell activation, affinity maturation, and the differentiation of GC B cells into memory and long-lived plasma cells (Fig. 3; Breitfeld et al., 2000; Schaerli et al., 2000; Vinuesa et al., 2016). Therefore, optimizing T cell help is an additional leverage point to improving the quality and longevity of humoral immune responses. In humans, circulating T_fh_ (cT_fh_) cells, which emigrate from lymphoid tissues, functionally and phenotypically resemble lymphoid T_fh_ cells and, hence, are an attractive alternative for studying T_fh_ cell responses in vaccine trials (Morita et al., 2011; Schmitt et al., 2014). Indeed, CSP-specific cT_fh_ cell frequencies correlated with protection in a recent RTS,S trial, highlighting the potential relevance of antigen-specific cT_fh_ cells for vaccine efficacy (Pallikkuth et al., 2020).

Influenced by the inflammatory milieu, cT_fh_ cells can be biased to a T helper type 1 (Th1)-, Th2-, or Th17-like phenotype (cT_fh_1, cT_fh_2, and cT_fh_17 cells) associated with differences in cytokine production that affect B cell isotype switching as well as differences in the overall capacity to provide B cell help, which is stronger for cT_fh_2 and cT_fh_17 cells compared with cT_fh_1 cells (Morita et al., 2011). In natural *Pf* malaria infection and experimental human *Pf* blood-stage infection, where the cytokine milieu is dominated by IFN-γ, cT_fh_1 cells are induced predominantly and have been linked to impaired B cell activation and differentiation into antibody-secreting cells in vitro (Obeng-Adjei et al., 2015; Chan et al., 2020). However, the relevance of cT_fh_1 cells in the *Pf* immune response in vivo is poorly understood. Vaccine vectors and adjuvants affect the cytokine milieu and can be used to modulate the quantity, as well as the quality, of cT_fh_ cells (Reed et al., 2013; Hill et al., 2019; Nielsen et al., 2021). The relevance of this for vaccine design has been recently demonstrated in two vaccine studies reporting that protein vaccines, targeting PfCSP or the merozoite protein PfRH5, supplemented with AS01 induced cT_fh_1 and cT_fh_2 responses associated with high antigen-specific antibody titers, whereas administration with a viral-vectored vaccine resulted in a pronounced cT_fh_1 skewing and reduced antibody titers (Bowyer et al., 2018; Nielsen et al., 2021). The data highlight that the optimization of vaccine compositions is a rational approach for enhancing T_fh_ responses. However, changes in the cytokine milieu alter the immune response beyond T_fh_ cells, and the relevance of the antibody response compared with IFN-γ–mediated immune orchestration for protection is still unclear (Perez-Mazliah and Langhorne, 2015). Further investigations of the role of these immune responses in protection are required to define the optimal vaccine composition.

For the development of a highly effective PfCSP vaccine, the identification of optimal T_fh_ cell targets along with antibody targets is critical. An ideal immunogen should contain conserved universal PfCSP epitopes, which are efficiently presented by a variety of MHC-II alleles and, hence, are not sensitive to geographic differences in parasite populations or MHC-II haplotype frequencies. So far, all studies that investigated PfCSP epitope targeting by CD4^+^ T cells did not discriminate between T_fh_ and non-T_fh_ cells. CD4^+^ T cells epitopes within PfCSP have been described in the junction between the N-terminus and central repeat region, as well as in the C-terminal domain (Fig. 2 B). Epitopes covering the repeating NANP motifs have not been reported, potentially due to their highly disordered structure, which might interfere with peptide presentation on MHC-II (Guy et al., 2015).

The most immunodominant and immunoprevalent epitopes comprise the overlapping epitopes Th2R and T*, which were shown to efficiently induce T cell responses in natural infections (Good et al., 1988; Guttinger et al., 1988; Sinigaglia et al., 1988a) and after sporozoite (Moreno et al., 1991) or PfCSP subunit (Lalvani et al., 1999; Schwenk et al., 2011) vaccination. Through TCR expression cloning from single cT_fh_ cells, we recently defined the epitope preferences of cT_fh_ cells to aa 311–333 (PSDKHIKEYLNKIQNSLSTEWSP) within the Th2R/T* region, which was independent of the haplotype (Wahl et al., 2021 *Preprint*). The second immunodominant epitope (CS.T3) in the C-terminal domain is located adjacent to the GPI anchor and is conserved among *Pf* isolates (Sinigaglia et al., 1988a; Lalvani et al., 1999; Schwenk et al., 2011). Both epitope regions are associated with broad MHC-II binding patterns and are hence classified as universal T cell epitopes (Guttinger et al., 1988; Sinigaglia et al., 1988a, 1988b; Moreno et al., 1991, 1993; Calvo-Calle et al., 1997). Another conserved epitope (T1) is located in the junctional region between the N-terminus and the central repeat region, covering NANP and NVDP motifs (Nardin et al., 1989). However, due to presentation on a restricted number of MHC-II alleles, T cell responses targeting the T1 epitope are infrequent, impeding vaccine development (Nardin et al., 1989; Calvo-Calle et al., 1997).

The strong dominance of the universally presented Th2R/T* region, which is highly polymorphic across different *Pf* isolates, might be one reason for the reduced protection against heterologous infections seen in RTS,S (Neafsey et al., 2015). Sequence variations have been shown to affect binding of the peptides to the MHC-II or TCR and, hence, prevent T cell activation, limiting the ability to respond to heterologous infections (Guttinger et al., 1988; Moreno et al., 1993; Zevering et al., 1994; Lalvani et al., 1999; Parra-López et al., 2006; Wahl et al., 2021 *Preprint*). Therefore, boosting of T_fh_ responses by natural infections might be impaired. Future efforts should investigate whether natural boosting of T_fh_ cells is required to maintain protective antibody titers or whether the induction of a robust T_fh_ response by vaccination is sufficient to induce long-lasting B cell responses.

In addition to PfCSP-specific T_fh_ cells, T_fh_ cells targeting the HBsAg carrier protein correlate with protection in RTS,S, although to a lesser extent than PfCSP-specific cT_fh_ cells (Pallikkuth et al., 2020), presumably due to the fact that HBsAg-specific T_fh_ cells can only provide help to PfCSP-specific B cells that ingested the HbsAg–PfCSP fusion protein. Whether the absence of non-PfCSP–coupled HBsAg in R21 compared with RTS,S improves the PfCSP-specific B cell help by HBsAg-specific T_fh_ cells and thereby humoral immunity and protection needs to be addressed.

## Concluding remarks

A hallmark of the humoral immune system is the ability to improve the quality of the response through affinity maturation. RTS,S induces high but short-lived antibody titers that correlate with protection. However, whether better and more durable protection can be achieved by inducing higher-quality responses remains to be determined. To address this question, a major goal in PfCSP vaccine development must be to increase the overall affinity and longevity of the B cell response against the protective antibody epitopes. The monoclonal analysis of the anti-PfCSP B cell response in humans has shown that high-affinity antibodies against the central repeat and the junctional region, which is not included in RTS,S or R21, can mediate protection and has defined the highly immunogenic C-terminus as a nonprotective target on live sporozoites. Affinity maturation and the formation of long-lived humoral memory relies critically on the formation of GC reactions and help from T_fh_ cells. Studies tracking T_fh_ cell activation and expansion on the monoclonal level appear essential for vaccine design strategies that aim at inducing optimal T_fh_ responses. The challenge will be to identify conserved PfCSP target epitopes that enable natural boosting and mediate the efficient induction of long-lived protective humoral immune responses. Alternatively, T cell help could be provided by universal non-PfCSP epitopes. Ideally, our efforts to understand the molecular and cellular mechanisms underlying vaccine formulation studies will then have to identify immunogen-adjuvant combinations, dosing regimens, and immunization schedules that optimally balance cellular and humoral immunity, including Th1 and Th2 responses, as well as antibody effector functions to achieve the highest efficacy. The fact that no clear correlate of protection has been identified suggests that a deeper mechanistic understanding of the role of individual immune cell subsets, and more importantly their interplay at the molecular level and with the parasite, seems to be necessary for the rational design of novel vaccine candidates against PfCSP and other *Pf* vaccine targets. The coming years will have to show whether vaccine strategies that aim at promoting the quality of anti-PfCSP responses by including optimized antibody and TCR target epitopes result in high efficacy independently of preexisting malaria immunity. These efforts should not be limited to adults given that the major target population will be infants.
